# Adherence to multidisciplinary care in a prospective chronic kidney disease cohort is associated with better outcomes

**DOI:** 10.1371/journal.pone.0266617

**Published:** 2022-10-14

**Authors:** Pablo Rios, Laura Sola, Alejandro Ferreiro, Ricardo Silvariño, Verónica Lamadrid, Laura Ceretta, Liliana Gadola, on behalf of all nephrologists who report to Programa de Salud Renal Uruguay Registry

**Affiliations:** 1 Comisión Asesora de Programa de Salud Renal, Fondo Nacional de Recursos, Montevideo, Uruguay; 2 Departamento de Nefrología, Facultad de Medicina, Universidad de la República, Montevideo, Uruguay; University of Sao Paulo Medical School, BRAZIL

## Abstract

**Introduction:**

The Renal Healthcare Program Uruguay (NRHP-UY) is a national, multidisciplinary program that provides care to chronic kidney disease (CKD) patients. In this study, we report the global results of CKD patient outcomes and a comparison between those treated at the NRHP-UY Units, with those patients who were initially included in the program but did not adhere to follow up.

**Methods:**

A cohort of not-on dialysis CKD patients included prospectively in the NRHP-UY between October 1^st^ 2004 and September 30^th^ 2017 was followed-up until September 30^th^ 2019. Two groups were compared: a) **Nephrocare Group:** Patients who had at least one clinic visit during the first year on NRHP-UY (n = 11174) and b) **Non-adherent Group**: Patients who were informed and accepted to be included but had no subsequent data registered after admission (n = 3485). The study was approved by the Ethics Committee and all patients signed an informed consent. Outcomes were studied with Logistic and Cox´s regression analysis, Fine and Gray competitive risk and propensity-score matching tests.

**Results:**

14659 patients were analyzed, median age 70 (60–77) years, 56.9% male. The Nephrocare Group showed improved achievement of therapeutic goals, ESKD was more frequent (HR 2.081, CI 95%1.722–2.514) as planned kidney replacement therapy (KRT) start (OR 2.494, CI95% 1.591–3.910), but mortality and the combined event (death and ESKD) were less frequent (HR 0.671, CI95% 0.628–0.717 and 0.777, CI95% 0.731–0.827) (p = 0.000) compared to the Non-adherent group. Results were similar in the propensity-matched group: ESKD (HR 2.041, CI95% 1.643–2.534); planned kidney replacement therapy (KRT) start (OR 2.191, CI95% 1.322–3.631) death (HR 0.692, CI95% 0.637–0.753); combined event (HR 0.801, CI95% 0.742–0.865) (p = 0.000).

**Conclusion:**

Multidisciplinary care within the NRHP-UY is associated with timely initiation of KRT and lower mortality in single outcomes, combined analysis, and propensity-matched analysis.

## Introduction

Chronic kidney disease (CKD) affects 10–20% of the population, increases the risk of cardiovascular events and death, and constitutes a significant public health problem [1–3]. Epidemiological studies demonstrate that therapeutic interventions on lifestyle and medication slow disease progression [4–6]. Uruguay is a South American country (3525000 inhabitants, area of 176215 Km^2^, Human Development Index (2019) 0.82) where health care has been universally available since 1911, and kidney replacement treatment (KRT) for End Stage Kidney Disease (ESKD) has been available for the entire population since 1980. The country has a National Integrated Health System (*Sistema Nacional Integrado de Salud* or *SNIS*) [https://www.impo.com.uy/bases/leyes/18211-2007/61] made up of a network of public and non-for-profit private providers. Renin-angiotensin system blockers (RASB), statins and alkali oral buffer therapy are widely available throughout the country. Since 2004 a National Renal Healthcare Program [7, 8] (NRHP-UY) was developed to promote medical and public education on kidney diseases, and to incorporate kidney healthcare at the primary care level, to improve prevention and early CKD diagnosis and treatment, including therapeutic education and personalized follow-up by a multidisciplinary team [http://www.fnr.gub.uy/home_psaludrenal].

### Study aims

The main aim is to analyze the outcomes of a not-on dialysis CKD patient cohort cared within a multidisciplinary health care program (the NRHP-UY), and compare the adherent with the non-adherent patients’ outcomes.

## Methods

The NRHP-UY Registry held by the National Resources Fund (NRF) and the Uruguayan Renal Healthcare Program Advisory Committee (Comisión Asesora de Salud Renal, CASR), includes patients with CKD diagnosis, defined as an estimated glomerular filtration rate (eGFR) less than 60 ml/min/1.73m^2^ and/or proteinuria ≥ 150 mg/day (or albuminuria ≥ 30 mg/day in diabetics) for at least 3 months, who are voluntarily included in the CKD Registry [http://www.fnr.gub.uy]. Therapeutic goals are clearly defined and annually monitored [http://www.fnr.gub.uy/home_psaludrenal]. The NRHP-UY Advisory Committee coordinates multidisciplinary consensus to elaborate national evidence-based clinical practice guidelines, that are periodically updated, freely accessible online and also distributed free of charge in printed versions [7–11]. Also, coordinates activities to public health education and multiple nationwide workshops on CKD diagnosis and treatment, to primary level staff.

Participant healthcare institutions (public and private) sign an Agreement with the NRF by which they ensure the care of not-on dialysis CKD outpatients by a team that includes a nephrologist (in all clinic visits), a nutritionist, a nurse, a social worker and a psychologist (the NRHP-UY Unit) so CKD diagnosis is always confirmed by a nephrologist. A specific software ("Sistema María ") generates online registration of users admitted to the NRHP-UY and manages notices (“alarms”) that are sent by the NFR to the NRHP-UY Unit staff when a patient does not comply with the studies and clinic visits expected according to the CKD stage. In that case, the Unit staff telephones those patients and coordinates a clinic visit; it is the patient’s choice whether to attend or not. [http://www.fnr.gub.uy/sites/default/files/programas/convenio_marco_asse.pdf]. Patients are referred to multidisciplinary nephrological care by primary level physicians, nephrologists or other medical specialties. Patients are included voluntarily and sign an informed consent to allow registration of their data. Multidisciplinary teams have been developed nationwide in the 50 Health care provider institutions with NRHP-UY Units, and data are registered (NRHP-UY Registry) on the specific centralized software. Therapeutic education is provided by the Unit staff [9–11] with the goals of encouraging patient lifestyle changes (e.g., the cessation of smoking; increase exercise; adjust the intake of salt and protein and weight loss, if appropriate) and to use a multidrug approach to slow or stabilize CKD progression and timely KRT initiation if necessary [7, 8, 12–16]. The patients are periodically checked on their clinical status and biochemical parameters by a nephrologist, according to CKD stage. The Nutritionists evaluated them at inclusion and periodically, provided nutritional personalized counseling and specific diet prescriptions. The Nurses promote adherence to treatment, resolve the “alarms” sent from the NFR, by contacting patients, on a case-management style, as well as psychologists and social workers, if required.

Serum creatinine is measured with a national standardized method as recommended [17] and a consensus on the determination of albuminuria/proteinuria is conducted with consistent methods by every laboratory in the country [18, 19]. The NRHP-UY Advisory Committee coordinates the Clinical Practice Guidelines for the Diagnosis and Treatment of CKD based on national consensus [9–11] according to international recommendations [20–23] and collects individual patients’ data on online forms. The NRHP-UY Registry resides in the Data Center of the National Resources Fund (NRF) and is confidentially cross-referenced with the mandatory National Registry of chronic Kidney Replacement Therapy (KRT) and with the Death Registry of the Ministry of Health (all-cause mortality). In order to achieve a homogeneous, fair care for all patients, annual evaluations of the NRHP-UY units are regularly conducted. The 50 NRHP-UY Units cover up to 74.8% of the country population. The general and specific objectives, detailed implementation of main strategies and the preliminary results of the NRHP-UY have been described elsewhere [7, 8, 12–16].

In this study, longitudinal data was analyzed for a CKD adult patient cohort (over 15 years old), prospectively admitted to the NRHP-UY registry between October 1^st^ 2004 and September 30^th^ 2017, followed-up until September 30^th^ 2019.

**Fig 1:**
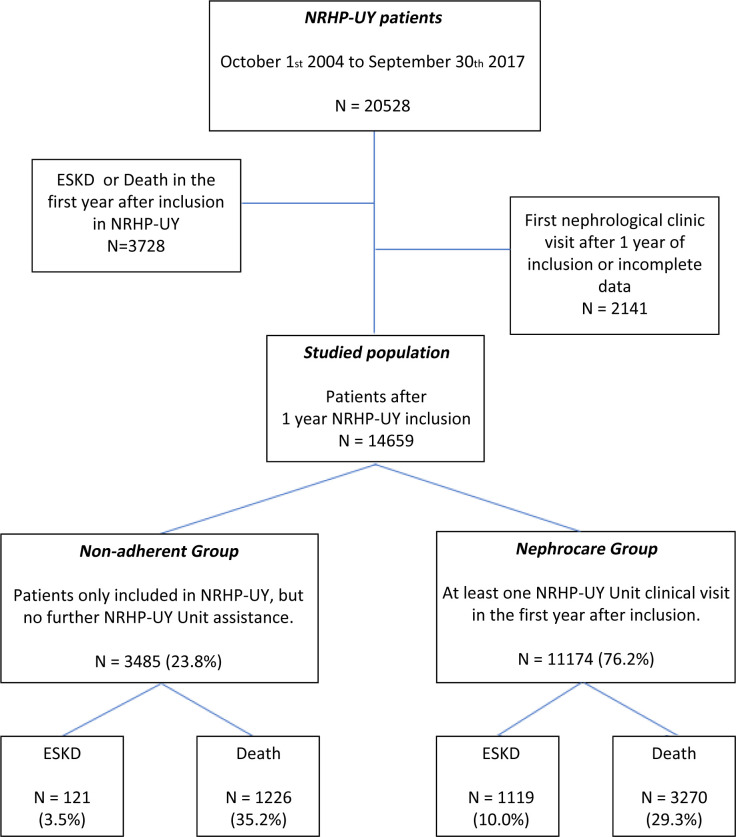
Algorithm of selection criteria. Total population in the National Renal HealthCare Program Uruguay (NRHP-UY), the studied groups distribution and the evolution with follow-up until September 30th 2019.

The cohort inclusion criteria were: a) meeting the admission criteria for the NRHP-UY, and b) having at least one year of death-free and KRT-free survival between the date of registration to the NRHP-UY and the inclusion in the study. Patients who attended the first nephrology clinic visit beyond 1 year of inclusion (poor adherence) or had incomplete baseline data were excluded from the studied cohort and subsequent analysis (Fig 1).

Patients included (n = 14659) were divided into two groups based on assistance to clinic visits: a) **Nephrocare group**: patients with baseline data and at least one registered clinic visit during the first year on NRHP-UY and subsequent years (n = 11174) and b) **Non-adherent group:** patients with baseline data registry at first (and only) clinic visit but no subsequent clinic visit registered after the initial one, although may continue under non-NRHP-UY medical care (n = 3485). Every person in the country has access to medical care, even free of charge, so the Non-adherent group may be assisted by other colleagues, even nephrologists, as some of them eventually received KRT. The Nephrocare group was assisted to by the multidisciplinary NRHP-UY Units (as described) and their clinic visit data were properly registered. All deaths in the country are recorded in the Death Registry of the Ministry of Health and the mandatory Registry of Chronic KRT of the NRF includes every person receiving chronic dialysis or renal transplantation across the country, the initial modality (hemodialysis-HD- or peritoneal dialysis-PD-), the vascular access to first hemodialysis (arterio-venous fistula -AVF-, permanent or transitory central venous catheter -CVC-) and the circumstances of dialysis initiation (planned or urgent start) as the attending nephrologists stated in the chronic KRT start official authorization form. In both studied groups, if a patient was not admitted to KRT or died (as for the mentioned mandatory national registries), that patient was deemed alive by September 30^th^ 2019.

Age, sex, nephropathy, comorbidities (diabetes, hypertension, smoking habits, ischemic heart disease, chronic heart failure, peripheral arteriopathy and/or stroke), healthcare provider and laboratory and clinic data are prospectively registered upon admission to the NRHP-UY and at the last clinic visit: body mass index (BMI), systolic and diastolic blood pressure (SBP/DBP), proteinuria, serum creatinine, phosphatemia, hemoglobin, venous bicarbonate (when data available), glycated hemoglobin A1c in diabetics, lipid profile, estimated glomerular filtration rate by CKD-EPI formula, treatment with renin–angiotensin–aldosterone system blockers (RASB), statins, buffer/binding agents and/or erythropoietin. Deaths and KRT initiation were actively monitored until September 30^th^ 2019. Initial data and final outcome (death and KRT) were available in both groups, but by the inclusion criteria, clinic visit data were only available in the Nephrocare group.

Operational definitions [4, 9–11]: Body mass index (BMI) was calculated as weight (Kg) divided by square height (m^2^). Diabetes was defined as serum fasting glucose ≥126 mg/dl, non-fasting glucose ≥200 mg/dl, glycated hemoglobin A1c ≥6.5%, use of antidiabetic drugs or by self-report on medical records. Cardiovascular disease was defined as history of coronary artery disease, prior revascularization, heart failure, stroke or peripheral arterial disease. Smoking status as current smoking (last 6 months) self-reported. Hypertension was defined as systolic blood pressure ≥140 and/or diastolic blood pressure ≥90 mmHg or use of anti-hypertensive drugs. Blood pressure registered was office measured. Achievement of goals for metabolic targets and risk factors included: blood pressure (individualized target below 140/90 mmHg), urine protein <500 mg/day (or urine protein/creatinine ratio <500 mg/g), phosphatemia < 4.6 mg/dl, venous bicarbonate ≥ 23 mEq/l, total cholesterol < 200 mg/dl, triglycerides <150 mg/dl, hemoglobin over 12 g/dl (women) and 13 g/dl (men) in CKD stages I to III and over 10.6 g/dl in CKD stages IV-V (but below 11,5 g/dl if the patient was taking erythropoietin), Glycated hemoglobin (HbA1c) individualized, between 6,5–8% in diabetics [23], non-smoking as reported in the data recorded by attending nephrologist. Obesity: if BMI was ≥30 kg/m^2^. Estimated glomerular filtration rate was calculated per CKD-EPI Formula [6] (creatinine measures are standardized to Isotope Dilution Mass Spectrometry (IDMS) traceable methods all-over the country) and CKD stages were defined by K-DIGO 2012 [4, 5]. Proteinuria was measured following National [10] and International Recommendations [4, 5] and categorized: a) urine protein <500 mg/day or urine protein-creatinine ratio < 500 mg/g b) urine protein ≥500 mg/day or urine protein-creatinine ratio ≥500 mg/g. Renin–angiotensin–aldosterone system blockers (RASB) and statins treatments are registered: a) at registration to the NRHP-UY (in both groups) and b) during the clinic visits in the Nephrocare group, as well as buffer agents. End Stage Kidney Disease (ESKD) is defined by KRT initiation.

Healthcare provider (public or non-for-profit private) was also registered, as a surrogate marker of socio-economic level, as persons with a monthly income less than USD 1900 are assisted at public institutions free of charge. [https://www.asse.com.uy/contenido/Mision-y-Vision-2113].

The primary outcomes measured are initiation of maintenance dialysis or receipt of a kidney transplant (KRT) or all-cause death. Secondary outcomes (only in the Nephrocare group) are improvement on the achievement of clinical targets between the first and the last clinic visit registered.

### Statistical analysis

For the descriptive analysis, data is presented as summary measures (median and interquartile range, percentage and confidence interval with 95% dispersion). Tests adjusted to variable nature and distribution were used for the statistical inference analysis (Mann Whitney, Chi^2^, Wilcoxon, McNemar, Poison and Kaplan-Meier tests). Risk estimation was conducted by calculating the odds ratio (OR) by logistic regression or the hazard ratio (HR) by multivariate Cox’s regression, with the corresponding 95% confidence interval (CI). Fine and Gray competitive risk analysis adjusted to covariates were performed to ESKD risk estimation. Propensity score matching was used to match subjects in both groups in terms of age, sex, CKD stage, diabetes, proteinuria, hypertension, cardiovascular comorbidities (ischemic heart disease, heart failure, stroke and/or peripheral arterial disease) and healthcare provider as a surrogate marker of patient incomes, (match 1:1, caliper 0.1) and then risk analysis were performed by logistic and Cox´s regression.

In every case, the null hypothesis is rejected at p < 0.05 or with overlapping 95% confidence intervals. IBM SPSS 15.0, STATA 16.0 and R version 4.0.3 (2020-10-10) software were used for the analysis.

#### Ethics

Patient included in the NRHP-UY Registry signed an Informed Consent. Pursuant to Law No. 18331 (*Habeas Data*) and as to maintain confidentiality, no identifying sensitive data was included in the databases. The systematic analysis of data from the NRHP-UY was approved by the Ethics Committee of the Faculty of Medicine of the University of the Republic. (09/25/2006) (File N° 071140-002077-06).

This study adhered to the guidelines by the STROBE group for cohort observational studies [24, 25].

## Results

### Population

Since October 1^st^, 2004 to September 30^th^ 2017, 20655 patients were admitted to the NRHP-UY, and 14659 accomplished inclusion criteria (Fig 1).

They were 56.9% men with median age of 70 (60–77) years and 34.5% were cared at public institutions. On admission to NRHP-UY proteinuria was absent or below 500 mg/day in 85.1%, median systolic blood pressure was 130 (120–140) mmHg, median diastolic blood pressure 80 (70–80) mmHg and median estimated glomerular filtration rate 40.89 (30.74–53.13) ml/min/1.73m^2^. The most frequent nephropathies were vascular (47.8%), diabetic (13%), tubulointerstitial nephropathies (5.5%) and glomerulopathies (4.5%) (Table 1). In this cohort, at admission to NRHP-UY, 715 (8.4%) men and 275 (4.4%) women smoked (overall 990, 6.8%) (Chi^2^ p = 0.000).

**Table 1:** Global population baseline characteristics.

	Non-adherent Group	Nephrocare Group	Global population	p
Number	3485	11174	14659	
Age (years) (median, pc 25–75)	71.0 (60.0–78.0)	70.0 (61.0–77.0)	70.0 (60.0–77.0)	0.015^a^
Age groups: ≤ 40 years, n (%)	230 (6.6%)	705 (6.3%)	935 (6.4%)	0.540^b^
41–64 years n (%)	931 (26.7%)	3083 (27.6%)	4014 (27.4%)	0.539 ^b^
≥65 years n (%)	2324 (66.7%)	7386 (66.1%)	9710 (66.2%)	0.628 ^b^
Sex Male, n (%)	2048 (58.8%)	6296 (56.3%)	8344 (56.9%)	0.012 ^b^
***Health provider***				0.000 ^b^
Public n (%)	1317 (37.8%)	3743 (33.5%)	5060 (34.5%)	
Private-Non-for-profit n(%)	2168 (62.2%)	7431 (66.5%)	9599 (65.5%)	
SBP initial (mmHg) (median, pc 25–75)	130 (120–140)	130 (120–140)	130 (120–140)	0.020 ^b^
DBP initial (mmHg) (median, pc 25–75)	80 (70–80)	80 (70–80)	80 (70–80)	0.850 ^b^
***Nefropathies***				
Vascular, n (%)	1731 (49.9%)	5256 (47.2%)	6987 (47.8%)	0.000 ^b^
Diabetics, n (%)	517 (14.9%)	1383 (12.4%)	1900 (13.0%)	
Obstructive Tubulo-intersticial, n (%)	216 (6.2%)	581 (5.2%)	797 (5.5%)	
Glomerulopathies, n (%)	102 (2.9%)	553 (5.0%)	655 (4.5%)	
***Cardiovascular risk factors and Comorbidities***				
Arterial Hypertension, n (%)	3027 (86.9%)	9828 (88.0%)	12855 (87.7%)	0.086 ^b^
Diabetes, n (%)	1404 (40.3%)	4049 (36.2%)	5453 (37.2%)	0.000 ^b^
Smoking, n (%)	251 (7.2%)	739 (6.6%)	990 (6.8%)	0.230 ^b^
Obesity, n (%)	1098 (39.0%)	3480 (39.1%)	4578 (39.1%)	0.913 ^b^
Ischaemic heart disease, n (%)	715 (20.5%)	2152 (19.3%)	2867 (19.6%)	0.106 ^b^
Stroke, n (%)	174 (5.0%)	542 (4.9%)	716 (4.9%)	0.722 ^b^
Heart failure, n (%)	317 (9.1%)	830 (7.4%)	1147 (7.8%)	0.002 ^b^
Lower limbs artery disease, n (%)	188 (5.4%)	537 (4.8%)	725 (4.9%)	0.165 ^b^
eGFR initial (ml/min/1.73 m^2^) (median, pc 25–75)	44.37 (33.51–57.52)	39.94 (29.91–51.67)	40.89 (30.74–53.13)	0.000^a^
***CKD stages at inclusion***				
I, n (%)	277 (7.9%)	692 (6.2%)	969 (6.6%)	0.230 ^b^
II, n (%)	491 (14.1%)	1053 (9.4%)	1544 (10.5%)	
III, n (%)	2121 (60.9%)	6604 (59.1%)	8725 (59.5%)	
IV, n (%)	521 (14.9%)	2498 (22.4%)	3019 (20.6%)	
V, n (%)	75 (2.2%)	327 (2.9%)	402 (2.7%)	
***Proteinuria***				
No proteinuria	2438 (70.0%)	7778 (69.6%)	10216 (69.7%)	0.253 ^b^
<500 mg/day (or PCR< 500 mg/g), n (%)	554 (15.9%)	1700 (15.2%)	2254 (15.4%)	
≥500 mg/day (or PCR≥500 mg/g), n (%)	493 (14.1%)	1696 (15.2%)	2189 (14.9%)	
***Initial treatment***				
RASB n (%)	2028 (58.2.%)	6958 (62.3%)	8986 (61.3%)	0.000 ^b^

CV = Cardiovascular comorbidities # = ischemic heart disease, chronic heart failure, peripheral arteriopathy and/or stroke.

SBP = Systolic blood pressure, DBP = Diastolic blood pressure, PCR = urine protein-creatinine ratio, RASB = Renin-angiotensin system blockade.

Statistics: ^a^Test Mann Whitney,

^b^ test Chi^2^.

At admission to the NRHP-UY, patients presented frequent vascular risk factors and comorbidities as hypertension (87.7%), diabetes (37.2%) and obesity (39.1%) (Table 1).

Most patients had no proteinuria and the CKD diagnosis was based on low eGFR. CKD stages [4, 5] corresponded to stage III in 59.5% of cases and to stage IV in 20.6% of cases (Table 1).

At admission, both groups showed differences (Table 1). Median age and initial eGFR were higher for the Non-adherent group (71 *vs* 70 years and 44.37 *vs* 39.94 ml/min/1.73m^2^). Vascular nephropathy and comorbidities such as diabetes and heart failure were more frequent in the Non-adherent group, whereas a RASB treatment at admission was more frequent amongst the Nephrocare group (62.3% *vs* 58.2%) (Table 1).

As some baseline characteristics of both groups are significantly different (Table 1) a propensity score matching was done, (1:1, caliper 0.1), on sex, age, diabetes, hypertension, proteinuria, CKD stage, cardiovascular comorbidities, healthcare provider type and RASB treatment. The matched groups’ baseline characteristics are shown on Table 2.

**Table 2:** Matched population baseline characteristics.

	Non-adherent Group	Nephrocare Group	Total matched	p
Number	3480	3480	6960	
Age (years) (median, pc 25–75)	71.0 (60.0–78.0)	70.0 (60.0–77.0)	70.0 (60.0–78.0)	0.020^a^
Age groups: ≤ 40 years, n (%)	229 (6.6%)	229 (6.6%)	458 (6.6%)	
41–64 years n (%)	929 (26.7%)	951 (27.3%)	1880 (27.0%)	0.834 ^b^
>65 years n (%)	2322 (66.7%)	2300 (66.1%)	4622 (66.4%%)	
Sex Male, n (%)	2044 (58.7%)	2000 (57.5%)	4044 (58.1%)	0.296 ^b^
***Health provider***				0.552 ^b^
Public n (%)	1313 (37.7%)	1288 (37.0%)	2601 (37.4%)	
Private-Non-for-profit n (%)	2167 (62.3%)	2192 (63.0%)	4359 (62.6%)	
SBP initial (mmHg) (median, pc 25–75)	130 (120–140)	130 (120–140)	130 (120–140)	0.684 ^b^
DBP initial (mmHg) (median, pc 25–75)	80.0 (70.0–80.0)	78.5 (70.0–80.0)	80.0 (70.0–80.0)	0.122 ^b^
***Nefropathies***				0.001 ^b^
Vascular, n (%)	1730 (49.9%)	1573 (45.4%)	3303 (47.7%)	
Diabetics, n (%)	514 (14.8%)	493 (14.2%)	1007 (14.5%)	
Obstructive Tubulo-intersticial, n (%)	216 (6.2%)	172 (5.0%)	388 (5.6%)	
Glomerulopathies, n (%)	101 (2.9%)	164 (4.7%)	265 (3.8%)	
***Cardiovascular risk factors and Comorbidities***				
Arterial Hypertension, n (%)	3023 (86.9%)	3003 (86.3%)	6026 (86.6%)	0.504 ^b^
Diabetes, n (%)	1399 (40.2%)	1396 (40.1%)	2795 (40.2%)	0.961 ^b^
Smoking, n (%)	250 (7.2%)	242 (7.0%)	492 (7.1%)	0.743 ^b^
Obesity, n (%)	1095 (38.9%)	1080 (38.9%)	2175 (38.9%)	1.000 ^b^
CV comorbidities, # n (%)	1113 (32.0%)	1100 (31.6%)	2213 (31.8%)	0.757 ^b^
eGFR initial (ml/min/1.73 m^2^) (median, pc 25–75)	44.32 (33.51–57.44)	42.78 (33.02–56.86)	43.49 (33.33–57.23)	0.024^a^
***CKD stages at inclusion***				
I, n (%)	273 (7.8%)	317 (9.1%)	590 (8.5%)	0.230 ^b^
II, n (%)	490 (14.1%)	459 (13.2%)	949 (13.6%)	
III, n (%)	2121 (60.9%)	2105 (60.5%)	4226 (60.7%)	
IV, n (%)	521 (15.0%)	536 (15.4%)	1057 (15.2%)	
V, n (%)	75 (2.2%)	63 (1.8%)	138 (2.0%)	
***Proteinuria***				
No proteinuria	2434 (69.9%)	2422 (69.6%)	4856 (69.8%)	0.892 ^b^
<500 mg/day, n (%)	553 (15.9%)	551 (15.8%)	1104 (15.9%)	
≥500 mg/day, n (%)	493 (14.2%)	507 (14.6%)	1000 (14.4%)	
***Initial treatment***				
RASB n (%)	2026 (58.3%)	2056 (59.1%)	4082 (58.6%)	0.480 ^b^

CV = Cardiovascular comorbidities # = ischemic heart disease, chronic heart failure, peripheral arteriopathy and/or stroke.

SBP = Systolic blood pressure, DBP = Diastolic blood pressure, PCR = urine protein-creatinine ratio, RASB = Renin-angiotensin system blockade.

Statistics: ^a^Test Mann Whitney,

t ^b^ est Chi^2^.

### Nephrocare group clinical-biochemical data and treatment (Tables 3 and 4)

**Table 3:** Global Nephrocare group baseline and final data.

	First visit	Last visit	p
Blood Pressure <140/90 mmHg, n (%) (n = 11086)	6248 (56,4)	7103 (64.1)	0.000^a^
Hemoglobin (g/dl) median, pc 25–75 (n = 8093)	12.8 (11.6–14.0)	12.7 (11.5–14.0)	0.000 ^b^
Hemoglobin ≥10,6 g/dl (in CKD IV-VND) n (%) (n = 2171)	1687 (77.7)	1739 (80.1)	0.028^a^
Total Cholesterol (mg/dl) median, pc 25–75 (n = 6405)	192 (162–226)	176 (149–206)	0.000 ^b^
Total Cholesterol <200 mg/dl, n (%) (n = 6405)	3567 (55.7)	4463 (69.7)	0.000 ^a^
TG (mg/dl) median, pc 25–75 (n = 5858)	142 (102–200)	128 (94–178)	0.000 ^b^
TG < 150 mg/dl, n (%) (n = 5858)	3167 (54.1)	3643 (62.2)	0.000 ^a^
BMI (kg/m^2^) median, pc 25–75 (n = 8623)	28.5 (25.2–32.2)	28.2 (25.0–32.3)	0.000 ^b^
BMI < 30 (kg/m^2^) n (%) (n = 8623)	5252 (60.9)	5334 (61.9)	0.016 ^a^
Plasma Albumin (g/dl) median pc 25–75 (n = 1882)	4.2 (3.9–4.5)	4.2 (3.9–4.4)	0.334 ^b^
Plasma Albumin ≥ 4 g/dl, n (%) (n = 1882)	1314 (69.8)	1281 (68.1)	0.180 ^a^
Phosphatemia (mg/dl) median, pc 25–75 (n = 2228)	3.7 (3.3–4.2)	3.7 (3.2–4.2)	0.005 ^b^
Phosphatemia < 4.6 mg/dl n (%) (n = 2259)	1886 (84.2)	1914 (85.4)	0.194 ^a^
PTH (pg/ml) median, pc 25–75 (n 2421)	No data	109 (64–203)	--
PTH < 70 (pg/ml) n (%) (n = 2421)	No data	675 (27.9)	--
Venous bicarbonate (mEq/l) median, pc 25–75 (n = 3917)	No data	24 (22–26)	--
Venous bicarbonate ≥ 23 mEq/l n (%) (n = 3917)	No data	2732 (69.7)	--
Non-smoking n (%) (n = 11174)	10435 (93.4)	10879 (97.4)	0.000 ^a^

Median Time in-between 43 (18–76) months. Number of annual clinic visits: median (pc 25–75) 1.31 (0.77–1.97) visit /patient-year. (n = available paired-data in each variable). There are not PTH or Venous bicarbonate data at First visit. ND = not on dialysis, TG = Triglycerides, BMI = Body mass index, PTH = Parathyroid hormone.

^a^ McNemar’s paired samples test,

^b^ Wilcoxon test.

**Table 4:** Global Nephrocare group treatments.

	First visit	NRHP-UY assistance	p
RASB, n (%) *(n = 11174)*	6960 (62.3)	7906 (70.7)	0.000^a^
Statins, n (%) *(n = 11174)*	4457 (39.9)	6431 (57.5)	0.000^a^
Erythropoietin use, n (%) (n = 2825)^b^	0	505 (17.9)	NC
Buffer agents, n (%) (n = 2556) ^b^	0	663 (25.9)	NC
Influenza vaccine, n (%) (n = 5912)	881 (14.7)	3045 (51.5)	0.000^a^
Pneumococcal vaccine, n (%) (n = 5912)	403 (6.8)	2204 (37.3)	0.000^a^
Hepatitis B vaccine, n (%) (5912)	91 (1.5)	750 (12.7)	0.000^a^

RASB = Renin-angiotensin system blockade, ^b^ in CKD IV-VND stages, n = number of patients with available paired-data in each variable.

^a^Mc Nemar’s paired samples test.

Median number of clinic visits registered per patient was 5 (pc25-75: 2–9) and 1.31 (0.77–1.97) visit /patient-year. In the Nephrocare population (n = 11174), analysis of paired data between the first and last data showed significant improvement in several of the data analyzed. Improvement in blood pressure, lipids, hemoglobin targets on erythropoietin in CKD IV-V, and lesser smoking and obesity were achieved. Venous bicarbonate data were not available at inclusion and partially on evolution, and of those 69.7% were ≥ 23 mEq/L in the last data registered, under diet and buffer agents (25.9% of CKD stages IV-V). The percentage of patients treated with statins significantly increased when compared to admission to NRHP-UY (39.9% *vs* 57.5%), as did RASB treatment (62.3% *vs* 70.7%), and vaccination (e.g. Influenza 14.7% *vs* 51.5%) (p = 0.000). Besides, 17.9% of patients with initial CKD stage IV-V received erythropoietin.

### ESKD and death in Nephrocare *vs* Non-adherent group

End Stage Kidney Disease (ESKD) rate (1.47 ESKD/100 patients-year in global population) is higher in the Nephrocare group *vs* Non-adherent group (1.68 *vs* 0.66 /100 patients-year) and they started KRT with a higher eGFR (9.3 *vs* 8.2 ml/min/1.73m^2^), more frequently planned (44.2% *vs* 21.2%), and those on HD more frequently used AVF as their first vascular access (Table 5). The risk of ESKD (requirement for KRT) (adjusted to age, sex, diabetes, blood pressure, proteinuria, smoking, CKD stage, cardiovascular comorbidities and RASB initial treatment) was significantly higher in the Nephrocare Group (HR 2.081, CI 95% 1.722–2.514) (Tables 5 and S1). Also, in the Nephrocare *vs* Non-adherent group the chance of a planned vs urgent KRT start was higher (OR 2.494, CI 95% 1.591–3.910) adjusted to age, sex, diabetes, cardiovascular comorbidities and eGFR at KRT star (covariates that may influence KRT initiation circumstances). Global population death rate was 5.32/100 patients-year, significantly lower in the Nephrocare group *vs* Non-adherent group (4.92 *vs* 6.74/100 patients-year) (p < 0.05) (Tables 5 and S1–S4).

**Table 5:** Outcome analysis. Exposition time, ESKD and death incidence rates. Adjusted multivariate Cox regression (adjusted to age, sex, diabetes, blood pressure, proteinuria, smoking, CKD stage, cardiovascular comorbidities and RASB initial treatment) in all patients.

All population	Non-adherent Group	Nephrocare Group	Global population	Test
Death, n (%)	1226 (35.2%)	3270 (29.3%)	4496 (30.7%)	
ESKD, n (%)	121 (3.5%)	1119 (10.0%)	1240 (8.5%)	
KRT Modality: HD-PD, n (%)	112 (92.6%)- 9 (7.4%)	969 (86.6%)-150 (13.4%)	1081 (87.2%)-159 (12.8%)	p = 0.621^a^
KRT planned start, n (%)	25 (21.2%) (3 no data)	468 (44.2%) (61 no data)	493 (41.9%) (64 no data)	p<0.0001^a^
AVF used at 1^st^ HD	10 (11.1%) (22 no data)	218 (31.9%) (285 no data)	228 (29.5%) (307 no data)	p<0.0001^a^
Death + ESKD, n (%)	1347 (38.6%)	4389 (39.3%)	5736 (39.1%)	
Follow-up time (sum) (years-patient)	18179	66392	84572	
Follow-up time *(days) (median*, *pc25-75)*	1624 (1010–2687)	2008 (1221–2965)	1919 (1165–2916)	
eGFR-KRT *(ml/min/1*.*73m*^*2*^*) (median*, *pc25-75)*	8.18 (5.9–10.9)	9.32 (7.3–11.9)	9.32 (7.13–11.79	p = 0.026^b^
ESKD rate (Events/100 patient-year)	0.66	1.68	1.47	p<0.05^c^
Death rate (Events/100 patient-year)	6.74	4.92	5.32	p<0.05^c^
Death+ ESKD rate (Events/100 pt-year)	7.40	6.61	6.78	p<0.05^c^
**Statistical model**	**Event**	**Groups contrasted**	**HR / OR (IC 95%)**	**p**
Cox Regression^d^	Death	Nephrocare *vs* Non-adherent (Ref)	HR 0.671 (0.628–0.717)	0.000
Cox Regression^d^	ESKD	Nephrocare *vs* Non-adherent (Ref)	HR 2.081 (1.722–2.514)	0.000
Logistic Regression^e^	Planned KRT start^f^	Nephrocare *vs* Non-adherent (Ref)	OR 2.494 (1.591–3.910)	0.000
Cox Regression^d^	ESKD+Death	Nephrocare *vs* Non-adherent (Ref)	HR 0.777 (0.731–0.827)	0.000
Fine and Gray (competitive risk)	ESKD (death censored)	Nephrocare *vs* Non-adherent (Ref)	HR 2.447 (1.969–3.042)	0.000

^a^Chi^2^,

^b^Mann-Whitney,

^c^Poison tests,

^d^Cox regression, adjusted to age, sex, diabetes, blood pressure, smoking, initial CKD stage, proteinuria, cardiovascular comorbidities and RASB therapy,

^e^Logistic regression adjusted to age, sex, diabetes, CV comorbidities and eGFR at KRT start.

CKD = Chronic Kidney Disease, eGFR-KRT = estimated Glomerular filtration rate at chronic kidney replacement treatment start, ESKD = End Stage Kidney Disease, HD = Hemodialysis, PD = Peritoneal dialysis, AVF = Arterio-venous fistula, RASB = Renin-angiotensin system blockade.

^f^as stated by the attending nephrologists at chronic KRT start official authorization form.

By Fine and Gray competitive risk analysis of ESKD, censored by death, the Nephrocare group has a significantly higher risk (HR 2.447, CI 95% 1.969–3.042) *vs* the Non-adherent group in the global as well as in the matched population (HR 2.358, CI 95% 1.868–2.976) (Table 6). Death risk was significantly increased in men, diabetics, proteinuric, who smoked and had cardiovascular comorbidities, and higher ESKD stage (S2 Table). Death risk was significantly lower with initial RASB treatment and in the Nephrocare group, independent of risk factors (Cox’s multivariate regression adjusted HR 0.671, CI 95% 0.628–0.717). The risk of a combined event of ESKD/death in the Nephrocare group was significantly lower than in the Non-adherent group (Cox’s regression multivariate adjusted HR 0.777, CI 95% 0.731–0.827) (S3 Table).

**Table 6:** Outcome analysis. Exposition time, ESKD and death incidence rates. Adjusted multivariate Cox regression (adjusted to age, sex, diabetes, blood pressure, proteinuria, smoking, CKD stage, cardiovascular comorbidities and RASB initial treatment) in matched population.

**Matched population**	**Non-Nephrocare Group**	**Nephrocare Group**	**Total matched population**	**Test**
n	3480	3480	6960	
Death, n (%)	1224 (35.2%)	1032 (29.6%)	2256 (32.4%)	
ESKD, n (%)	121 (3.5%)	273 (7.8%)	394 (5.7%)	
KRT Modality: HD-PD, n (%)	112 (92.6%)- 9 (7.4%)	242 (88.6%)-31 (11.4%)	354 (89.8%)-40 (10.2%)	p = 0.234^a^
KRT planned initiation§, n (%)	25 (21.2%) (3 no data)	101(38.9%) (14 no data)	126 (33.4%) (17 no data)	p<0.001 ^a^
AVF used at 1^st^ HD	10 (11.1%) (22 no data)	49 (27.5%) (64 no data)	59 (22.0%) (86 no data)	p<0.001 ^a^
Death + ESKD, n (%)	1345 (38.6%)	1305 (37.5%)	2650 (38.0%)	
Follow-up time (sum) (years-patient)	18134	20934	39068	
Follow-up time *(days) (median*, *pc25-75)*	1621 (1010–2686)	2027 (1241–2999)	1821 (1121–2874)	
eGFR_KRT *(ml/min/1*.*73m*^*2*^*) (median*, *pc25-75)*	8.18 (5.92–10.99)	9.33 (7.31–12.32)	9,03 (6,73–11,96)	p = 0.000 ^b^
ESKD rate (Events/100 patient-year)	0.67	1.30	1.01	p < 0.05^c^
Death rate (Events/100 patient-year)	6.75	4.93	5.77	p < 0.05 ^c^
Death+ ESKD rate (Events/100 pt-year)	7.42	6.23	6.78	p < 0.05 ^c^
**Statistical model**	**Event**	**Groups contrasted**	**HR / OR (IC 95%)**	p
Cox Regression ^d^	Death	Nephrocare *vs* Non-adherent (Ref)	HR 0.692 (0.637–0.753)	0.000
Cox Regression ^d^	ESKD	Nephrocare *vs* Non-adherent (Ref)	HR 2.041 (1.643–2.534)	0.000
Logistic Regression ^e^	Planned KRT start^f^	Nephrocare *vs* Non-adherent (Ref))	OR 2.191 (1.322–3.631)	0.002
Cox Regression ^d^	ESKD+Death	Nephrocare *vs* Non-adherent (Ref)	HR 0.801 (0.742–0.865)	0.000
Fine and Gray (competitive risk)	ESKD (death censored)	Nephrocare *vs* Non-adherent (Ref)	HR 2.358 (1.868–2.976)	0.000

^a^Chi^2^,

^b^ Mann-Whitney,

^c^ Poison tests,

^d^Cox regression, adjusted to age, sex, diabetes, blood pressure, smoking, initial CKD stage, proteinuria, cardiovascular comorbidities and RASB therapy,

^e^Logistic regression adjusted to age, sex, diabetes, CV comorbidities and eGFR_KRT.

CKD = Chronic Kidney Disease, eGFR_KRT = estimated Glomerular filtration rate at kidney replacement treatment start, ESKD = End Stage Kidney Disease, HD = Hemodialysis, PD = Peritoneal dialysis, AVF = Arterio-venous fistula, RASB = Renin-angiotensin system blockade.

^f^as stated by the attending nephrologists at chronic KRT start official authorization form.

Death causes data were only available until December 31^st^ 2016: 2217 in the Nephrocare group (34.5% cardiovascular, 21.4% malignancies,10.0% infectious and 3.2% kidney failure) and 722 in the Non-adherent group (33.4% cardiovascular, 20.6% malignancies, 8.2% infectious and 2.9% kidney failure).

The matched Nephrocare group also showed a higher ESKD rate (1.30 *vs* 0.67/100 patients-year) and ESKD risk (HR 2.041, CI95% 1.643–2.534) by multivariate adjusted Cox regression, a lower death rate (4.93 *vs* 6.75/100 patients-year) and risk (HR:0.692, CI95% 0.637–0.753) and lower combined event rate (6.23 *vs* 7.42/100 patients-year) and risk (HR 0.801, CI95% 0.742–0.865) (Tables 6 and S1–S3) similar to those observed in the global population. Also, in the matched groups analysis the chance of a planned KRT start was higher in the Nephrocare *vs* Non-adherent group (OR 2.191, CI 95% 1.322–3.631) by multivariate logistic regression adjusted to age, sex, diabetes, cardiovascular comorbidities and eGFR at KRT start (Tables 6 and S1–S4).

**Fig 2:**
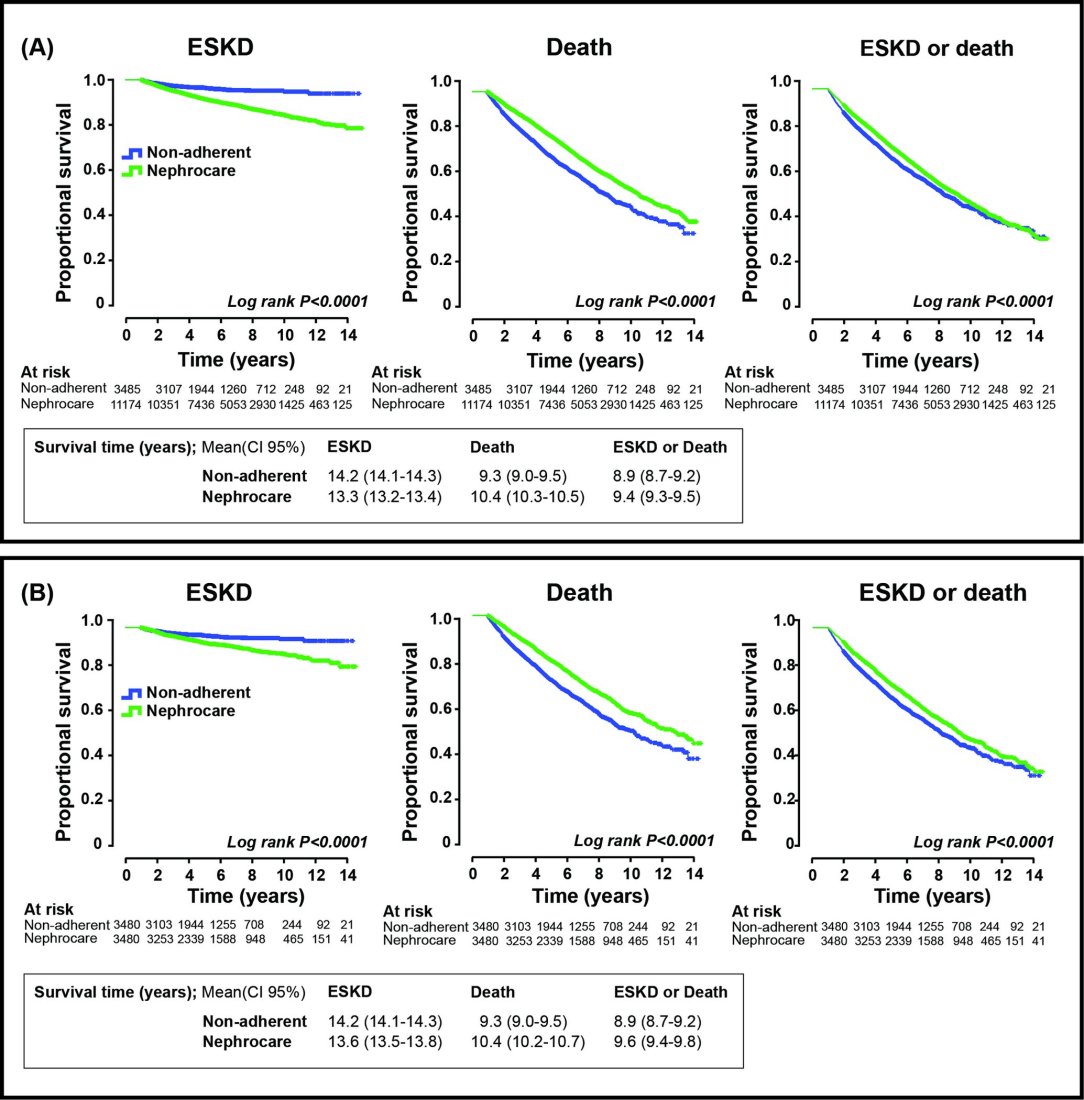
Survival curves. Survival curves (Kaplan-Meier) to End stage kidney disease (ESKD), death or both (combined event). A. Global population. B. Matched population.

Mean survival time to combined event were significantly longer in the Nephrocare *vs* Non-adherent groups: 9.394 (CI 95% 9.275–9.513) *vs* 8.948 (CI 95% 8.714–9.181) years (global population) and 9.638 (CI 95% 9.425–9.851) *vs* 8.946 (CI 95% 8.712–9.179) years (matched groups) (Fig 2).

## Discussion

The main goal of this study was to evaluate the impact of the NRHP-UY in CKD patients’ outcome. NRHP-UY is a nationwide effort that covers up 74.8% of country population, in which institutions as well as each single patient freely choose to participate. The NRHP-UY cohort has a death rate (5.32/100 patients-year) comparable with other reports [26, 27]. As 24% of patients are initially diagnosed and registered, but not furthermore assisted by NRHP-UY multidisciplinary teams, we wonder if, that initial nephrological visit was enough or, a further assistance by NRHP-UY teams would improve CKD patients’ outcomes.

The question is: did the multidisciplinary care provided by the NRHP-UY Units obtain significantly different outcomes compared to the usual care of CKD patients? In order to answer that, the NRHP-UY cohort was retrospectively divided in two groups: those who participated in programmed clinic visits (Nephrocare group) with those who were registered (as they were cared for at institutions participants on NRHP-UY, fulfilled inclusion criteria and signed inform consent), but afterwards did not attend NRHP-UY clinic visits, and therefore did not receive the multidisciplinary care offered due to unknown reasons. The selection of a non-adherent group for comparison may introduce bias, as non-adherent patients’ outcome is reported to be worst [28], but it has the advantage that their baseline data are available in the NRHP-UY Registry (so they have similar origin) and their final outcome data are available in the mandatory national registries of KRT and death.

In this study, the Nephrocare group achieved diminished mortality and a lower combined death/ ESKD rate despite a higher ESKD rate. The NRHP-UY included multidisciplinary care teams (leaded by nephrologists), as utilized in other countries with outstanding results [29–32]. In recent years, the importance of prospective cohort studies in the analysis of CKD [33–36] has been highlighted as they show the “real world” data. The large NRHP-UY cohort started more than 15 years ago provides such evidence.

### Characteristics of the population studied (Tables 1 and 2)

CKD patients studied showed a predominance of men (56.9%), that was higher than the overall Uruguayan population (47% men in the country’s population ≥ 15 years) [37]. and they were predominantly elderly (Table 1), as is in other cohort studies of CKD patients [32–36, 38–40]. The studied groups had other differences (Table 1), so a multivariate analysis and propensity score matched groups were obtained (Table 2). In spite of that, the matched groups still had differences: the Non-adherent patients were slightly older and their underlying nephropathies were different (e.g. glomerulopathies were less frequent). As these facts may impact on outcome, the matched population Cox regression analysis were also adjusted to all covariates, including age, nephropathies and eGFR)

### Clinical and biochemical data in the Nephrocare group (Tables 3 and 4)

The Nephrocare population showed improvement in the achievement of most target values [39–61]. Nevertheless, although statistically significant (because it is a huge population) some data changes under NRHP-UY care may be not clinically relevant. Blood pressure target in CKD patients is under debate [41–43]. The SPRINT study [44, 45] was the first to include CKD patients and demonstrated that SBP under 120 mmHg increased patient survival. Nevertheless, it excluded diabetics and patients with eGFR below 20 ml/min/1.73m^2^, so its results have been questioned [41]. This study sets target blood pressure below 140/90 mmHg as that was the criterion used during the timeframe analyzed. Likewise, prescriptions for RASB treatment increased significantly from admission to last visit (Table 4). Anemia frequency increases as CKD progresses, as is well-known [51–55], but the percentage of patients with hemoglobin above 10.6 g/dl (in CKD stages IV-V) slightly increased in the Nephrocare group, and 17.9% received erythropoietin. Acidosis is frequent, despite diet and buffer agents prescribed. Acidosis is a known factor of CKD progression [56] and, in a previous national study of this cohort, it was observed that acidosis was associated with higher CKD progression and death [57], so this is a factor that NRHP-UY must improve. The prescription of statins among CKD population has been controversial, but the SHARP [58] study provided evidence favoring it. The NRHP-UY Guideline recommended it [10], a significant increase in statins prescription was observed and more patients improved lipid profile (Table 3). These improvements may result also from diet and/or a better adherence to prescribed medication achieved by therapeutic education provided by the multidisciplinary NRHP-UY teams: nephrologists, nurses, nutritionists, social workers and psychologists [9–11, 59, 60].

### Outcome (Tables 5 and 6 and S1–S4)

Data from this study confirm the association of known risk factors (age, male sex, diabetes, hypertension, smoking, cardiovascular comorbidities) and survival among the population studied both in multivariate Cox’s regression in the entire cohort and in the matched groups (Tables 5 and 6 and S1–S4). As socio-economic factors are also associated with poor outcomes [62, 63], the propensity score matching included health provider (https://www.asse.com.uy/contenido/Mision-y-Vision-2113) as a surrogate of patients’ incomes. NRHP-UY global population mortality rate (5.32 /100 patient-year) is comparable with international reports [26, 27] and is even lower in the Nephrocare group (4.92 vs 6.74/100 patient-year). Independently from other risk factors, the Nephrocare group has a lower death risk and a lower risk of the combined event (death or ESKD), in spite of a higher chance of reaching ESKD and being admitted to KRT, but with higher eGFR, more frequently planned, and, those on HD more frequently used an AVF as their first vascular access (Tables 5 and 6). The fact that ESKD/KRT had a higher risk among the Nephrocare group (in global and matched groups), even if death competitive risk is considered seems to contradict the improvement observed in the achievement of therapeutic goals (Tables 3 and 4). However, a lower mortality rate among this group makes it more likely for survivors to require KRT. As they are closely checked by a nephrological team and the central alarm system prevented missing scheduled check-ups, they may survive enough and a timely, planned chronic KRT admission is more likely, as it was observed. Other authors [64–71] observed a similar phenomenon. As Nicoll et al. [66] signaled most patients on CKD stages IV-V are asymptomatic, but have a higher risk of cardiovascular death or ESKD, so self-management (as may be the situation in the Non-adherent group) is probably insufficient. Therapeutic education, multidisciplinary care and a well-functioning alarm system may allow a timely, planned admission into chronic KRT and, probably, a lower mortality afterwards, as has been reported [72–76]. Also, healthcare staff and patients’ education (as the NRHP-UY provided) has been associated with planned KRT start and better outcomes [65, 77]. First HD vascular access via an AVF (more frequently observed in the Nephrocare group) is highly recommended [72], as it has been associated with less infectious complications and better outcomes. Besides, the incidence and prevalence of patients on KRT in the country (universally available) did not significantly increase in the last 15 years [78–80]. Finally, the combined risk of ESKD and death is significantly lower for the Nephrocare group, both in the global population (HR 0.777, p = 0.000) and in the matched groups (HR 0.801 p = 0.000) (Tables 5 and 6), both adjusted to covariates. In the present study all-cause mortality was the outcome analyzed, but the main cause of death observed was cardiovascular, as previously shown [18, 67].

### Study strengths and limitations

This study has limitations: 1) The data registry is non-mandatory, and nephrologists report data at different time intervals, 2) for the Non-adherent group, it is not possible to ensure if they have received unreported medical or nephrological care, or if they are non-adherent also to diet or medications, 3) participant nephrological teams may be more motivated, 4) there may be heterogeneities structure and function among NRHP-UY Units, as well as differences in patient referral by other physicians, in patient education strategies or promotion of adherence, on laboratory tests and on the recommended frequency of clinic visits. All these facts may introduce bias.

The study has several strengths: 1) This is a large cohort with a long follow-up of more than ten years, with hard outcome indicators [81] obtained as a result of multidisciplinary care and a centralized data collection, in the context of real-world healthcare. 2) The observation period allows for the detection of low frequency events and ensures that differences observed between both groups are not a random by-product of observer bias. 3) Data collection was conducted prospectively on-line in a format created for that purpose, with exact definitions for each variable to decrease variability in the collection of data reported. 4) Both groups present data obtained at the beginning of the observation period on biological variables, comorbidities, laboratory tests and treatments, allowing for a precise statistical alignment adjusted to determinant prognostic variables. 5) Outcome indicators are obtained from national mandatory registries.

## Conclusions

CKD patients were at a greater risk of death (5.32/100 patients-year) than to reach ESKD (1.47 ESKD/100 patients-year). The multidisciplinary renal healthcare program of Uruguay, directed to stabilize CKD progression in a large cohort of patients with a long follow-up, allowed for a timely KRT initiation and is associated with a significantly lower mortality.

## Supporting information

S1 Table End stage kidney disease (ESKD) risk. Cox regression multivariate analysis in global and matched population.
(PDF)

S2 Table Death risk. Cox regression multivariate analysis in global and matched population.
(PDF)

S3 Table Death and end stage kidney disease (ESKD) risk. Cox regression multivariate analysis in global and matched population.
(PDF)

S4 Table Planned kidney replacement treatment. Logistic regression analysis, adjusted to sex, age, diabetes, cardiovascular comorbidities and estimated glomerular filtration at kidney replacement treatment start, in global and matched population.
(PDF)

S1 Dataset NRHP-UY studied population. Data from patients who accomplished inclusion criteria.
(SAV)
