# Supplementary material for: Adherence to multidisciplinary care in a prospective chronic kidney disease cohort is associated with better outcomes
Source: PLoS One. 2022 Oct 14;17(10):e0266617. doi: 10.1371/journal.pone.0266617 (PMC9565398; doi:10.1371/journal.pone.0266617)
Supplement: S1 Table — Cox regression multivariate analysis in global and matched population. (PDF) [file pone.0266617.s001.pdf]

S1Table. **End stage kidney disease (ESKD) risk.** Cox regression multivariate analysis. adjusted to sex. age. diabetes. smoking. obesity. cardiovascular comorbidities, CKD stages, initial systolic and diastolic blood pressure, proteinuria, RASB initial treatment and studied groups, in global and matched population

**End stage kidney disease risk**		**Global population**			**Matched population**	
	HR	CI 95%	p	HR	CI 95%	p
Sex [Reference Men]	0.758	0.675-0.851	0.000	0.758	0.614-0.935	0.010
Age (year) [Continuous]	0.966	0.962-0.970	0.000	0.965	0.958-0.972	0.000
Diabetes [Reference No]	1.707	1.518-1.920	0.000	1.912	1.551-2.356	0.000
Systolic blood pressure (mmHg) [Continuous]	1.008	1.005-1.012	0.000	1.006	0.999-1.125	0.087
Diastolic blood pressure (mmHg) [Continuous]	0.996	0.990-1.002	0.150	1.002	0.991-1.013	0.739
Proteinuria (mg/day) [Reference <500 mg/day)]	3.216	2.843-3.638	0.000	2.853	2.286-3.561	0.000
Cardiovascular comorbidities [Reference No]	0.994	0.874-1.130	0.930	1.238	0.995-1.540	0.056
Smoking [Reference No]	1.281	1.065-1.541	0.008	1.572	1.159-2.133	0.004
CKD stage I [Reference eGFR ≥ 90 ml/min/1.73m^2]			0.000			0.000
Stage II (FG 89.9 .45ml/min/1.73m^2) [Ref Stage 1]	2.395	1.475-3.890	0.001	3.425	1.608-7.294	0.001
Stage III (FG 49.9-30 ml/min/1.73m^2) [Ref Stage 1]	6.896	4.509-10.545	0.000	8.326	4.142-16.734	0.000
Stage IV (FG 29.9-15 ml/min/1.73m^2) [Ref Stage 1]	39.116	25.609-59.747	0.000	51.800	25.787-104.053	0.000
Stage V (FG < 15 ml/min/1.73m^2) [Ref Stage 1]	67.188	43.279-104.304	0.000	88.006	42.725-181.279	0.000
RASB [Reference No]	0.889	0.792-0.998	0.046	0.795	0.646-0.978	0.030
**Nephrocare Group [Reference Non-adherent Group]**	**2.081**	**1.722-2.514**	**0.000**	**2.041**	**1.643-2.534**	**0.000**

CKD= Chronic kidney disease. RASB= Renin-angiotensin system blockade.

.
