# Supplementary material for: Adherence to multidisciplinary care in a prospective chronic kidney disease cohort is associated with better outcomes
Source: PLoS One. 2022 Oct 14;17(10):e0266617. doi: 10.1371/journal.pone.0266617 (PMC9565398; doi:10.1371/journal.pone.0266617)
Supplement: S3 Table — Cox regression multivariate analysis in global and matched population. (PDF) [file pone.0266617.s003.pdf]

S3 Table. **Death and End stage kidney disease (ESKD) risk.** Cox regression multivariate analysis, adjusted to sex, age, diabetes, smoking, cardiovascular comorbidities, CKD stages, initial systolic and diastolic blood pressure, proteinuria, RASB initial treatment and studied groups, in global and matched population.

**Death and ESKD (combined events) risk**		**Global population**			**Matched population**	
	HR	CI 95%	p	HR	CI 95%	p
Sex [Reference Men]	0.758	0.718-0.801	0.000	0.769	0.709-0.833	0.000
Age (year) [Continuous]	1.029	1.028-1.032	0.000	1.037	1.033-1.041	0.000
Diabetes [Reference No]	1.304	1.235-1.377	0.000	1.303	1.203-1.411	0.000
Systolic blood pressure (mmHg) [Continuous]	1.003	1.002-1.005	0.000	1.002	1.000-1.005	0.102
Diastolic blood pressure (mmHg) [Continuous]	0.996	0.993-0.999	0.011	0.998	0.994-1.002	0.314
Proteinuria (mg/day) [Reference <500 mg/day]	1.929	1.798-2.070	0.000	1.865	1.676-2.075	0.000
Cardiovascular comorbidities [Reference No]	1.332	1.261-1.407	0.000	1.394	1.288-1.509	0.000
Smoking [Reference No]	1.281	1.164-1.410	0.000	1.301	1.131-1.496	0.000
CKD stages [Reference I=eGFR ≥ 90 ml/min/1.73m^2]			0.000			0.000
Stage II (FG 89.9 .45ml/min/1.73m^2) [Ref Stage 1]	1.454	1.160-1.822	0.001	1.326	1.005-1.749	0.046
Stage III (FG 49.9-30 ml/min/1.73m^2) [Ref Stage 1]	2.495	2.027-3.071	0.000	2.122	1.641-2.744	0.000
Stage IV (FG 29.9-15 ml/min/1.73m^2) [Ref Stage 1]	5.103	4.126-6.310	0.000	4.138	3.165-5.411	0.000
Stage V (FG < 15 ml/min/1.73m^2) [Ref Stage 1]	8.021	6.340-10.149	0.000	5.927	4.301-8.168	0.000
RASB [Reference No]	0.866	0.821-0.914	0.000	0.851	0.787-0.921	0.000
**Nephrocare Group [Reference Non-adherent Group]**	**0.777**	**0.731-0.827**	**0.000**	**0.801**	**0.742-0.865**	**0.000**

CKD= Chronic kidney disease, RASB= Renin-angiotensin system blockade.
