# Supplementary material for: Adherence to multidisciplinary care in a prospective chronic kidney disease cohort is associated with better outcomes
Source: PLoS One. 2022 Oct 14;17(10):e0266617. doi: 10.1371/journal.pone.0266617 (PMC9565398; doi:10.1371/journal.pone.0266617)
Supplement: S4 Table — Logistic regression analysis, adjusted to sex, age, diabetes, cardiovascular comorbidities and estimated glomerular filtration at kidney replacement treatment start, in global and matched population. (PDF) [file pone.0266617.s004.pdf]

S4 Table. **Planned kidney replacement treatment.** Logistic regression analysis, adjusted to sex, age, diabetes, cardiovascular comorbidities and estimated glomerular filtration at kidney replacement treatment start, in global and matched population.

**Planned (*vs* urgent)* KRT start risk**	**Global population**			**Matched population**		
	OR	CI 95%	p	OR	CI 95%	p
Sex [Reference Men]	1.182	0.927-1.508	0.178	1.254	0.801-1.964	0.323
Age (year) [Continuous]	0.997	0.989-1.006	0.548	1.000	0.984-1.017	0.958
Diabetes [Reference No]	0.534	0.418-0.681	0.000	0.440	0.281-0.691	0.000
Cardiovascular comorbidities [Reference No]	0.810	0.615-1.066	0.132	0.864	0.529-1.411	0.558
eGFR_KRT (ml/min/1.73m^2) [Continuous]	1.042	1.019-1.065	0.000	1.029	0.996-1.063	0.082
**Nephrocare Group [Reference Non-adherent Group]**	**2.494**	**1.591-3.910**	**0.000**	**2.191**	**1.322-3.631**	**0.002**

* as stated by the attending nephrologist at chronic kidney replacement treatment (KRT) start in the official authorization form, eGFR_KRT= estimated glomerular filtration rate at kidney replacement treatment start.

CKD= Chronic kidney disease, RASB= Renin-angiotensin system blockade.

**Death and ESRD (combined events) risk**		**Global population**			**Matched population**	
	HR	CI 95%	p	HR	CI 95%	p
Sex [Reference Men]	0.758	0.718-0.801	0.000	0.769	0.709-0.833	0.000
Age (year) [Continuous]	1.029	1.028-1.032	0.000	1.037	1.033-1.041	0.000
Diabetes [Reference No]	1.304	1.235-1.377	0.000	1.303	1.203-1.411	0.000
Systolic blood pressure (mmHg) [Continuous]	1.003	1.002-1.005	0.000	1.002	1.000-1.005	0.102
Diastolic blood pressure (mmHg) [Continuous]	0.996	0.993-0.999	0.011	0.998	0.994-1.002	0.314
Proteinuria (mg/day) [Reference <500 mg/day]	1.929	1.798-2.070	0.000	1.865	1.676-2.075	0.000
Cardiovascular comorbidities [Reference No]	1.332	1.261-1.407	0.000	1.394	1.288-1.509	0.000
Smoking [Reference No]	1.281	1.164-1.410	0.000	1.301	1.131-1.496	0.000
CKD stages [Reference I=eGFR ≥ 90 ml/min/1.73m^2]			0.000			0.000
Stage II (FG 89.9 .45ml/min/1.73m^2) [Ref Stage 1]	1.454	1.160-1.822	0.001	1.326	1.005-1.749	0.046
Stage III (FG 49.9-30 ml/min/1.73m^2) [Ref Stage 1]	2.495	2.027-3.071	0.000	2.122	1.641-2.744	0.000
Stage IV (FG 29.9-15 ml/min/1.73m^2) [Ref Stage 1]	5.103	4.126-6.310	0.000	4.138	3.165-5.411	0.000
Stage V (FG < 15 ml/min/1.73m^2) [Ref Stage 1]	8.021	6.340-10.149	0.000	5.927	4.301-8.168	0.000
RASB [Reference No]	0.866	0.821-0.914	0.000	0.851	0.787-0.921	0.000
**Nephrocare Group [Reference Non-adherent Group]**	**0.777**	**0.731-0.827**	**0.000**	**0.801**	**0.742-0.865**	**0.000**
